# Transcranial Direct Current Stimulation May Reduce Prefrontal Recruitment During Dual Task Walking in Functionally Limited Older Adults – A Pilot Study

**DOI:** 10.3389/fnagi.2022.843122

**Published:** 2022-03-11

**Authors:** Azizah J. Jor’dan, Hagar Bernad-Elazari, Anat Mirelman, Natalia A. Gouskova, On-Yee Lo, Jeffrey M. Hausdorff, Brad Manor

**Affiliations:** ^1^Department of Exercise and Health Sciences, University of Massachusetts Boston, Boston, MA, United States; ^2^Hinda and Arthur Marcus Institute for Aging Research, Hebrew SeniorLife, Boston, MA, United States; ^3^Geriatric Research, Education, and Clinical Center, VA Boston Healthcare System, Boston, MA, United States; ^4^Harvard Medical School, Boston, MA, United States; ^5^Center for the Study of Movement, Cognition and Mobility, Neurological Institute, Tel Aviv Sourasky Medical Center, Tel Aviv, Israel; ^6^Sagol School of Neuroscience and Sackler Faculty of Medicine, Tel Aviv University, Tel Aviv, Israel; ^7^Division of Gerontology, Beth Israel Deaconess Medical Center, Boston, MA, United States; ^8^Rush Alzheimer’s Disease Center and Department of Orthopedic Surgery, Rush University Medical Center, Chicago, IL, United States

**Keywords:** oxygenated hemoglobin, gait, fNIRS, neural efficiency, brain stimulation

## Abstract

**Introduction:**

Transcranial direct current stimulation (tDCS) targeting the left dorsolateral prefrontal cortex (dlPFC) improves dual task walking in older adults, when tested just after stimulation. The acute effects of tDCS on the cortical physiology of walking, however, remains unknown.

**Methods:**

In a previous study, older adults with slow gait and executive dysfunction completed a dual task walking assessment before and after 20 min of tDCS targeting the left dlPFC or sham stimulation. In a subset of seven participants per group, functional near-infrared spectroscopy (fNIRS) was used to quantify left and right prefrontal recruitment defined as the oxygenated hemoglobin response to usual and dual task walking (ΔHbO2), as well as the absolute change in this metric from usual to dual task conditions (i.e., ΔHbO2_*cost*_). Paired *t*-tests examined pre- to post-stimulation differences in each fNIRS metric within each group.

**Results:**

The tDCS group exhibited pre- to post-stimulation reduction in left prefrontal ΔHbO2_*cost*_ (*p* = 0.03). This mitigation of dual task “cost” to prefrontal recruitment was induced primarily by a reduction in left prefrontal ΔHbO2 *specifically within the dual task condition* (*p* = 0.001), an effect that was observed in all seven participants within this group. Sham stimulation did not influence ΔHbO2_*cost*_ or ΔHbO2 in either walking condition (*p* > 0.35), and neither tDCS nor sham substantially influenced right prefrontal recruitment (*p* > 0.16).

**Discussion:**

This preliminary fNIRS data suggests that tDCS over the left dlPFC may modulate prefrontal recruitment, as reflected by a relative reduction in the oxygen consumption of this brain region in response to dual task walking.

## Introduction

Transcranial direct current stimulation (tDCS) is a non-invasive technique capable of safely and selectively modulating cortical excitability. A single, 20-min exposure to tDCS designed to facilitate the excitability of the left dorsolateral prefrontal cortex (dlPFC)—a region with known involvement in the complex control of gait (Jor’dan et al., 2017) as well as tasks that require working memory and verbal processing (Opitz et al., 2000)—improves the ability to maintain gait and balance control under dual task conditions, specifically, walking and standing while performing a serial subtraction cognitive task (Sohn et al., 2013; Zhou et al., 2014, 2021; Manor et al., 2016, 2018; Swank et al., 2016; Baharlouei et al., 2020; Dong et al., 2021; Mishra and Thrasher, 2021). These acute functional benefits of tDCS targeting the left dlPFC have been reported in healthy younger adults (Zhou et al., 2014), relatively healthy older adults (Manor et al., 2016; Zhou et al., 2021), older adults with mild impairments in gait and executive function (Manor et al., 2018), and in those with neurological conditions including Parkinson’s disease and stroke (Sohn et al., 2013; Swank et al., 2016; Baharlouei et al., 2020; Dong et al., 2021; Mishra and Thrasher, 2021). A recent meta-analysis of published evidence concluded that tDCS appears to be a promising tool to counteract age- and disease-related declines in gait and balance (Guo et al., 2020). Still, despite these observed benefits, the immediate after-effects of this form of non-invasive brain stimulation on cortical function during standing and walking remain largely unknown.

Functional near-infrared spectroscopy (fNIRS) is a wireless neuroimaging technique enabling the study of prefrontal recruitment *during* task execution. Specifically, fNIRS enables measurement of relative changes in oxygenated hemoglobin (HbO2), deoxygenated hemoglobin (Hb), and total hemoglobin concentration (THC) within local tissues during the performance of a given task (Menant et al., 2020). In younger adults (Mirelman et al., 2014), older adults (Holtzer et al., 2011, 2019), and in those with Parkinson’s disease (Nieuwhof et al., 2016) or cognitive impairment (Doi et al., 2013), walking while performing a mental arithmetic task, as compared to walking under normal conditions, increases prefrontal HbO2 concentration. Moreover, dual tasking appears to induce relatively greater increases in HbO2 concentration in older adults, as compared to younger adults (Udina et al., 2020). Together, these observations suggest that (1) dual task walking, as compared to usual walking, requires greater prefrontal recruitment during dual task performance (Doi et al., 2013; Nieuwhof et al., 2016), and that (2) aging may result in neural insufficiency (Stern, 2009; Holtzer et al., 2011) such that older as compared to younger adults require relatively greater recruitment of prefrontal resources in order to complete the dual task.

A single exposure of tDCS has been demonstrated to facilitate cortical excitability and functional connectivity, and importantly, decrease the blood oxygen level dependent signal of the targeted brain region(s) in response to the completion of cognitive tasks (Nissim et al., 2019; Abellaneda-Pérez et al., 2020). We therefore hypothesized that a single session of this type of tDCS would increase neural efficiency, that is, reduce the absolute change in prefrontal HbO2 concentration from usual walking to dual task walking. To test this hypothesis, we used fNIRS to examine prefrontal activation during a dual task walking assessment both before and immediately after a single 20-min session of tDCS targeting the left dlPFC or inactive sham stimulation.

## Materials and Methods

### Participants

Manor et al. (2018) completed a double-blinded, randomized, sham-controlled pilot trial studying the effects of a 10-day non-invasive brain stimulation intervention on gait, cognition, and mood in older adults with slow gait and mild-to-moderate executive dysfunction (NCT02436915). Fourteen participants were also willing to complete an additional protocol to test the immediate effects of their first assigned 20-min stimulation session on fNIRS-measured cortical activation during a dual task walking assessment. Inclusion criteria for the study included age ≥65 years, a preferred overground walking speed <1.0 m/s, and a Trail Making Test (TMT) B score below the 25th percentile of age- and education-based norms. Exclusion criteria included an inability to stand or ambulate unassisted, clinical history of stroke, Parkinson’s disease, or other physician-diagnosed neurological disorder, significant cognitive impairment defined by a Mini-Mental State Exam (MMSE) score ≤18, self-report of physician-diagnosed schizophrenia, bipolar disorder or other psychiatric illness, severe depressive symptoms as indicated by a Geriatric Depression Scale (GDS) score >11, or contradictions to tDCS including a report of seizure(s) within the past 2 years, use of neuro-active drugs, claustrophobia or risk of metal objects in the body, severe arthritis or lower-extremity pain, or physician-diagnosed peripheral neuropathy affecting the lower extremities. All participants provided written informed consent as approved by the Institutional Review Board.

### Protocol

#### Transcranial Direct Current Stimulation

**FIGURE 1:**
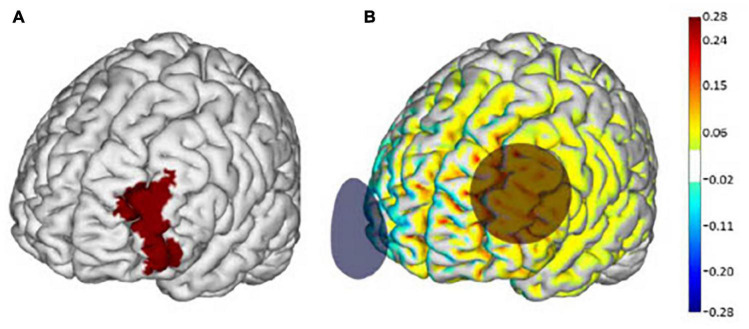
Transcranial direct current stimulation (tDCS) electrode placement and modeling of electrical current flow. **(A)** The left dorsolateral prefrontal cortex (dlPFC) (Brodmann Area 46) is highlighted in red. **(B)** Active tDCS was delivered at a maximum intensity of 2.0 mA with the anode (red circle) placed over the F3 regions and the cathode (blue circle) over the Fp2 region according to the 10–20 EEG placement system. Warmer and cooler colors depict the normal component of the electrical field produced by this stimulation, modeled on a standard brain as described in Miranda (2013). Images adapted from Manor et al. (2018) with permission from Neuroelectrics.

Participants were randomly assigned to receive either a 10-session tDCS or inactive sham (i.e., placebo) intervention. tDCS was delivered using the Starstim device (Neuroelectrics Inc., Barcelona, Spain) and was administered by experienced study personnel trained in the use of transcranial electrical stimulation. Participants and staff were blinded to the intervention arm using the blinding feature of the accompanying Starstim software program. Stimulation was delivered *via* two saline-soaked 35 cm^2^ synthetic sponges placed on the scalp. The anode (i.e., positive electrode) was placed over the F3 region of the 10–20 electroencephalography (EEG) electrode placement guide and the cathode (i.e., negative electrode) was placed over the right supra-orbital margin (Fp2) (Figure 1). tDCS stimulation was delivered at a max intensity of 2.0 mA continuously for 20 min. Sham stimulation consisted of the same montage and session duration. The sham current, however, was ramped up and then down to zero within 60 s of the stimulation start time. This approach is widely used as a control intervention and is suitable for blinding because scalp sensations arising from active tDCS typically occur at the beginning of application but fade within the first minute (Gandiga et al., 2006).

#### Walking Protocol

On the first day of the intervention, participants completed a walking paradigm immediately before and after the 20-min session of their assigned stimulation intervention. The paradigm consisted of three trials each of usual walking and dual task walking in randomized order over a 14-foot GAITRite mat (CIR Systems Inc., Havertown, PA, United States). Each walking trial consisted of two passes over the mat during one bout of continuous walking. Average gait speed (m/s) was computed by dividing the distance walked by time, which was quantified from each full step that occurred over the GAITRite mat within each trial. Gait speed was averaged within and then between trials.

Before the start of each walking trial, participants stood four feet before the mat for 60 s to enable baseline recording of fNIRS. Participants were then provided with standardized verbal instructions for the walking trial. For usual walking, participants were asked to walk at their “normal pace, as if they were walking down the sidewalk to go to the store and not in a hurry.” For dual task walking, participants were asked to walk, again at their normal pace, while verbally subtracting 3, 5, or 1 from a random three-digit number (as determined in a serial subtraction familiarization session in a prior visit). If the participant was unable to complete the serial subtraction task, participants were then instructed to walk at their normal pace, while verbally producing words that begin with a certain letter (i.e., verbal fluency task). Participants were encouraged to continue walking during the trial, and not stop, while performing their respective cognitive task, even if they forgot their place. If the participant forgot their place, they were instructed to continue from the last number/word response that they remembered. A rest period of at least 1 min was given after each trial.

#### Functional Near-Infrared Spectroscopy

Prefrontal cortex oxygenation was assessed using the Portalite^®^ fNIRS system (Artinis Medical System, Elst, Netherlands) to measure HbO2 during the performance of the walking assessment. Participants were instrumented with two separate pairs of near-infrared spectroscopy (NIRS) probes to monitor the absorption of light across the forehead. The NIRS transmitter and receiver pairs were placed over the left (Fp1) and right (Fp2) frontal cortex regions of the forehead, according to the modified international EEG 10–20 system at a height of 15% of the distance from nasion to inion and at 7% of the circumference from left and right. Positions of the transmitter and receiver were marked for each participant using a washable pen for re-instrumentation of the device after the stimulation intervention. The probes were attached to the skin using double-sided stickers and covered with a black cloth to prevent penetration of ambient light. fNIRS data were recorded continuously at 10 Hz, from the beginning of the quiet standing period to the end of the trial.

### Data Analysis

#### Prefrontal Cortex Oxygenation

The fNIRS device was equipped with a near-infrared (NIR) light source transmitter and a receiver placed on the surface of the skin (Menant et al., 2020). The Beer–Lambert law was used to calculate micromolar (μmol) changes in tissue oxygenation (i.e., HbO2) across time, using received optical densities from two continuous wavelengths of NIR light (760 and 850 nm). To eliminate physiologically irrelevant effects such as equipment noise, respiration, and heart pulsation from the raw data, a band-pass filter was applied with a finite impulse response filter, with a cut-off frequency between 0.01 and 0.14 Hz. We used the wavelet-based motion artifact removal proposed by Molavi and Dumont (2012), which is a channel-by-channel approach designed to correct for motion artifact. Additionally, we used correlation-based signal improvement (CBSI), which is a channel-by-channel approach developed by Cui et al. (2010) to reduce motion artifact caused by the movement of the head.

HbO2 concentrations (μmol/L) were assessed by hemisphere (left and right prefrontal region) after preprocessing of the raw data. HbO2 was selected as it is particularly sensitive to changes in cerebral blood flow and more reliable from trial to trial as compared to deoxygenated hemoglobin values (Miyai et al., 2001). Task-related changes in HbO2 concentration (ΔHbO2) were determined by subtracting average resting baseline HbO2 concentration before each task (i.e., during the last 20 s of quiet standing) from the average concentration during the active walking task (i.e., usual, or dual task walking) and averaging this value across similar trials (i.e., Prefrontal ΔHbO2 = Active walking HbO2 − Resting HbO2) (Chatterjee et al., 2019).

#### Dual Task Cost of Prefrontal Recruitment and Gait Speed

The dual task cost of prefrontal recruitment (i.e., ΔHbO2_*cost*_) was calculated as the absolute difference of changes in HbO2 concentration between dual task and usual walking (i.e., ΔHbO2_*dual task*_ – ΔHbO2_*usual*_) (μmol) (Chatterjee et al., 2019), separately by left and right hemisphere and within both pre- and post-stimulation assessments. The dual task cost to gait speed (m/s) was calculated as the absolute difference in gait speed between usual walking and dual task walking (i.e., Gait speed_*dual task*_ – Gait speed_*usual*_).

### Statistical Analysis

Student’s *t*-tests, Wilcoxon Rank Sum test, or Fisher’s Exact tests were used to compare group demographics and clinical characteristics, as appropriate. Paired *t*-tests were used to test the hypothesis that tDCS targeting the left dlPFC would decrease the left dual task cost of prefrontal recruitment (ΔHbO2_*cost*_). The brain activation (i.e., HbO2) and gait outcome measures at baseline (pre-stimulation) were paired with their respective measure post-stimulation for each participant. Pre- to post changes in the ΔHbO2 metrics were examined for normality graphically and by Shapiro–Wilk test and no significant departures from normality were noted. Linear regression was used to explore the relationships between tDCS-induced changes in prefrontal ΔHbO2 during dual task walking and the tDCS-induced change in dual task gait speed. The significance level was set at *p* < 0.05. In this pilot study, no mathematical correction was made for multiple comparisons. All analyses were performed using JMP software (SAS Institute, Cary, NC, United States).

## Results

### Participant Characteristics

All 14 participants completed the fNIRS study procedure and were included in the analysis (mean age 80 ± 11, 6 female). Seven of these participants were allocated to receive tDCS and seven were allocated to receive sham stimulation. Five (or 71%) tDCS and 6 (or 86%) sham participants completed the serial subtraction by 3s task. One sham participant completed the serial subtraction by 5s and two tDCS participants completed the verbal fluency task. One tDCS participant displayed atypical dual task gait speed (post-stimulation) and thus this data was excluded from the analysis. The tDCS and sham groups were similar in age, sex, BMI, handedness, usual and dual task gait speed, dual task cost to gait speed, and cognitive measures (i.e., MMSE and TMT B-A) (*p* > 0.10) (Table 1).

**TABLE 1:** Baseline demographic characteristics by cohort and group (tDCS vs. sham).

	Cohort	tDCS	Sham	*P*
*N*	14	7	7	
Age (years)	80 (11)	82 (11)	78 (11)	0.51
% Female	43	43	43	0.99
BMI	29.1 (4.6)	28.6 (4.5)	29.6 (5.0)	0.85
Handedness (% right)	66	85	50	0.27
Gait speed (m/s)				
Usual walking	0.68 (0.18)	0.71 (0.20)	0.65 (0.16)	0.56
Dual task waking	0.56 (0.20)	0.59 (0.28)	0.53 (0.13)	0.61
Dual task cost	−0.11 (0.10)^a^	−0.10 (0.07)^a^	−0.12 (0.12)^a^	0.73
Mini-Mental State Exam^‡^	26.5 (4)	24 (6)	27 (3)	0.10
TMT-B-A (s)	135.5 (78.1)	140.3 (84.5)	130.7 (77.7)	0.83

*Data = mean (SD); ^‡^ Data = median (interquartile range, Q3–Q1); TMT B-A = Trail Making Test Part B – Part A (seconds).*

*^a^Significant across and within group dual task change in gait speed compared to usual walking (p < 0.05).*

### The Effects of Walking on Prefrontal ΔHbO2 at Baseline (Pre-stimulation)

Within the pre-stimulation walking assessment, ΔHbO2 was similar across the left and right hemispheres for usual walking (left: 0.047 ± 0.089 μmol, right: 0.054 ± 0.104 μmol) and dual task walking (left: 0.145 ± 0.101 μmol, right: 0.134 ± 0.126 μmol) (*p* > 0.59). The ΔHbO2_*cost*_ (i.e., the absolute difference of changes in HbO2 concentration between dual task and usual walking) was also similar across left and right hemispheres (left: 0.098 ± 0.127 μmol, right: 0.080 ± 0.160 μmol) (*p* = 0.59).

The left prefrontal ΔHbO2 was greater for dual task walking as compared to usual walking (mean diff: 0.10 μmol, *p* = 0.01). A similar, but inconclusive trend was observed for right prefrontal ΔHbO2 (mean diff: 0.08 μmol, *p* = 0.08).

### The Effects of Transcranial Direct Current Stimulation on Prefrontal Recruitment During Walking

**FIGURE 2:**
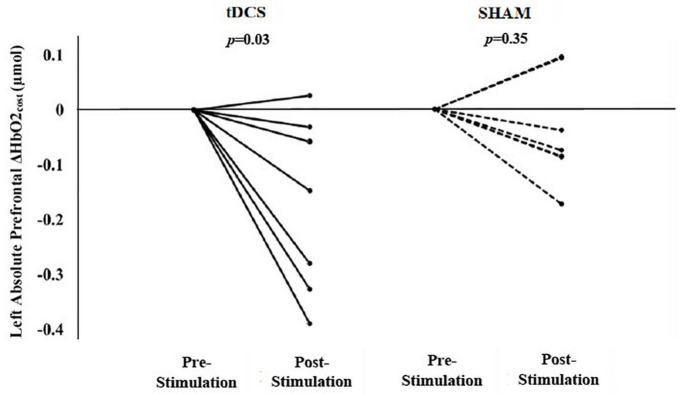
Participant-level data by group for the after-effects of a single session of tDCS or sham stimulation on left prefrontal Δ HbO2_*cost*_ (i.e., the absolute difference between dual task and usual walking). tDCS resulted in significant group changes in left Δ HbO2_*cost*_ (i.e., smaller left prefrontal HbO2 response from usual to dual task walking). Six of seven participants exhibited a reduction in left Δ HbO2_*cost*_. The sham stimulation resulted in insignificant group changes in left Δ HbO2_*cost*_. (Baseline data is normalized to zero where the post-intervention data reflects the absolute change in the left prefrontal ΔHbO2_*cost*_ from pre- to post-intervention.)

Within the tDCS group, there was a significant reduction in left prefrontal ΔHbO2_*cost*_ from pre- to post-stimulation (left mean diff: −0.17 μmol, *p* = 0.03). In fact, six out of seven participants exhibited a reduction in this metric following tDCS (Figure 2). The right prefrontal ΔHbO2_*cost*_ also appeared to decrease from pre- to post-stimulation; however, this change was not statistically significant (right mean diff: −0.12 μmol, *p* = 0.27). Within the sham group, there were no significant changes in left (Figure 2) or right prefrontal ΔHbO2_*cost*_ (mean diff <−0.04 μmol, *p* > 0.35).

**FIGURE 3:**
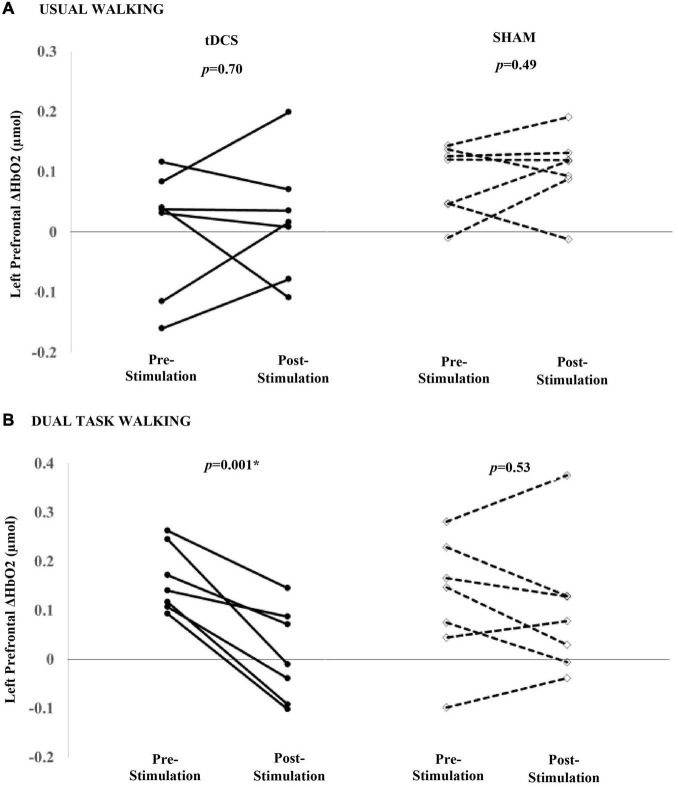
Participant-level effects of a single session of tDCS or sham stimulation on the left prefrontal ΔHbO2 during usual **(A)** and dual task walking **(B)**. tDCS nor sham was associated with a group-level change in left ΔHbO2 during usual walking. tDCS was associated with reduced left prefrontal ΔHbO2 during dual task walking. Seven of seven participants decreased left prefrontal ΔHbO2 during dual task walking. Sham was not associated with a significant decrease in left ΔHbO2.

Within the tDCS group, there were no significant group-level changes in left or right ΔHbO2 during usual walking (left mean diff: 0.02 μmol, *p* = 0.70; right mean diff: 0.01 μmol, *p* = 0.78). Similarly, sham stimulation did not result in changes in left or right prefrontal ΔHbO2 to usual walking (mean diff >0.02 μmol, *p* > 0.34) (Figure 3A). In contrast, left prefrontal ΔHbO2 in response to dual task walking was smaller after receiving tDCS (mean diff: −0.16 μmol, *p* = 0.001). All seven participants exhibited pre- to post reductions in left prefrontal ΔHbO2 during dual task walking (Figure 3B). A trend toward decreased right prefrontal ΔHbO2 was also observed at the group-level (mean diff: −0.11 μmol, *p* = 0.16). Within the sham group, there were no significant changes in left or right prefrontal ΔHbO2 to dual task walking (left mean diff: −0.02 μmol, *p* = 0.53; right mean diff: −0.02 μmol, *p* = 0.72).

### The Association Between Prefrontal ΔHbO2 and Walking Performance

At baseline (pre-stimulation), there were inconclusive correlations between the ΔHbO2 metrics and gait outcomes within the entire sample of 14 participants. All Pearson correlations were in the range of *r* = −0.11 to +0.15 (*p* > 0.63) except for the moderate associations between (1) right prefrontal ΔHbO2 and gait speed during usual walking (*r* = −0.33, *p* = 0.26), and (2) left prefrontal ΔHbO2_*cost*_ and the dual task cost to gait speed (*r* = +0.30, *p* = 0.33).

Pearson correlations between pre- and post-stimulation changes in ΔHbO2 metrics and changes in gait outcomes across and within groups, were also inconclusive, likely due to the small sample size. Across all participants, there were moderate, but statistically insignificant, correlations between pre- and post-stimulation change in (1) *left* prefrontal ΔHbO2 and gait speed during dual task walking (*r* = 0.30, *p* = 0.31) and (2) *left* prefrontal ΔHbO2_*cost*_ and dual task cost to gait speed (*r* = 0.31, *p* = 0.30). These moderate correlations were not present between the pre- and post-stimulation changes for *right* prefrontal ΔHbO2 metrics and gait outcomes (*r* = −0.001 to +0.16, *p* > 0.60) or *left* prefrontal ΔHbO2 and gait speed during usual walking (*r* = 0.25, *p* = 0.39).

## Discussion

In this pilot study, we sought to determine the potential effects of a single session of tDCS targeting the left dlPFC on walking-induced prefrontal HbO2 concentration in older adults with mild-to-moderate functional limitations. tDCS reduced left prefrontal ΔHbO2_*cost*_ at the group level, with six out of seven participants exhibiting a statistically significant reduction. Further analyses indicated that the tDCS-induced reduction in ΔHbO2_*cost*_ stemmed from relatively smaller increases in left prefrontal ΔHbO2 specifically within the dual task condition after stimulation. In contrast, tDCS targeting the left dlPFC did not influence right prefrontal ΔHbO2, and the sham group did not exhibit any changes in fNIRS metrics.

Transcranial direct current stimulation designed to facilitate the excitability of the left prefrontal cortex decreased the left (but not right) prefrontal ΔHbO2_*cost*_, as well as left prefrontal ΔHbO2 associated with dual task walking. In other words, participants were able to complete the dual task walking condition with a relatively smaller increase in left prefrontal activation after receiving tDCS. While the small sample size should be considered when generalizing the current observations, these data provide preliminary evidence suggesting that tDCS may facilitate prefrontal *neural efficiency* specifically as it pertains to dual task walking in older adults with mild-to-moderate functional limitations. The *neural efficiency theory* posits that brain activation adapts to the demands of the task being performed (Stern et al., 2012; Dunst et al., 2014). tDCS may have thus facilitated the ability to differentially recruit task-specific brain regions, thereby enabling less recruitment and/or neural processing to complete the dual task. In another study that used both fNIRS and tDCS stimulation, researchers reported that a single session of tDCS decreased ΔHbO2 during a memory task when tested just after stimulation (McKendrick et al., 2020). Additionally, Eggenberger et al. (2016) reported that the prefrontal cortical ΔHbO2 to walking was less following an 8-week exercise-based intervention as compared to baseline in older adults. The authors concluded that the exercise intervention facilitated a reduction in attention and other aspects of executive function, and thus prefrontal recruitment, needed to complete the dual task walking challenge. Since fNIRS is a *relative* measure that quantified the change between the standing and walking conditions, it is thus also plausible that results were influenced by tDCS-induced changes in HbO2 concentration during the standing trial that was used as a comparator to each walking trial. This possibility, however, would have likely affected both usual walking and dual task conditions similarly and, therefore, is less likely to have influenced the current results.

At baseline (i.e., pre-stimulation), left prefrontal ΔHbO2 was higher and walking speed was slower during dual task walking, as compared to usual walking. These results are aligned with numerous studies that have used fNIRS to demonstrate that dual task walking increases prefrontal blood oxygen levels (Holtzer et al., 2011; Doi et al., 2013; Mirelman et al., 2017). Although inconclusive, our results also indicated moderate associations between pre- and post-stimulation changes in left prefrontal ΔHbO2 and gait speed during dual task walking and the associated dual task costs across participants. Together these results suggest that dual tasking was associated with an increase in left—but not right—prefrontal ΔHbO2. Moderate associations between prefrontal activation and gait speed during dual tasking further suggests hemispheric specialization in the control of walking while performing a verbalized cognitive task (Doi et al., 2013; Fraser et al., 2016). Our results support the approach of using tDCS designed to facilitate the excitability of the left dlPFC to modulate cortical recruitment and mitigate dual task costs in older adults.

We focused our fNIRS recordings (and tDCS stimulation) on the prefrontal cortices as these regions are known be involved in the maintenance and control of gait. However, as studies have also implicated the involvement of additional regions (e.g., supplemental motor area and posterior parietal lobe), whole brain imaging is needed to understand the holistic effects of tDCS on brain functioning during dual task walking performance. For example, observed decreases in left prefrontal ΔHbO2_*cost*_ may have been accompanied by unmeasured increases in neural activation elsewhere in the brain. Secondly, we measured prefrontal recruitment (i.e., brain activation) using fNIRS *only*. The complementary use of ultrasound to measure blood flow in addition to the concentration of oxygenated hemoglobin (Csipo et al., 2021) would provide more complete neurophysiologic information regarding the effects of brain stimulation on brain activation during dual tasking. Thirdly, while blinding efficacy was assessed and achieved in the parent study following the completion of the entire intervention (i.e., 10 stimulation sessions) (Manor et al., 2018), participants’ beliefs in the type of stimulation received was not queried after the first stimulation session. Therefore, it is undetermined whether expectancy beliefs influenced the findings in the current results (Braga et al., 2021). Future studies should assess blinding efficacy after each stimulation visit in which outcome measures are taken. Lastly, the small sample size of this study may have led to under- or overestimation of the effects of tDCS and thus results should be interpreted with caution. Nevertheless, this pilot study indicates that tDCS may reduce prefrontal recruitment during dual task performance, suggesting that this form of non-invasive brain stimulation may increase neural efficiency of the prefrontal cortex. Larger, more definitive studies are, therefore, warranted to confirm these preliminary results.

## Data Availability Statement

The raw data supporting the conclusions of this article will be made available by the authors, without undue reservation.

## Ethics Statement

The studies involving human participants were reviewed and approved by the Hebrew SeniorLife Institutional Review Board. The patients/participants provided their written informed consent to participate in this study.

## Author Contributions

BM designed the study, obtained the funding, oversaw all aspects of data collection and analysis, and helped write the manuscript. AJ conducted the statistical analysis and data interpretation, and led the writing of this research. HB-E contributed to the processing of fMRI data and its interpretation. AM oversaw and contributed to the fMRI processing and its interpretation, statistical analysis, and manuscript preparation. O-YL assisted in the data collection and management, and manuscript preparation. NG and JH contributed to the statistical analysis, data interpretation, and manuscript preparation. All authors contributed to the article and approved the submitted version.

## Author Disclaimer

The content is solely the responsibility of the authors and does not necessarily represent the official views of Harvard Catalyst, Harvard University and its affiliated academic healthcare centers, the National Center for Research Resources, the University of Massachusetts Boston, or the National Institutes of Health.

## Conflict of Interest

The authors declare that the research was conducted in the absence of any commercial or financial relationships that could be construed as a potential conflict of interest.

## Publisher’s Note

All claims expressed in this article are solely those of the authors and do not necessarily represent those of their affiliated organizations, or those of the publisher, the editors and the reviewers. Any product that may be evaluated in this article, or claim that may be made by its manufacturer, is not guaranteed or endorsed by the publisher.
